# Randomized, placebo-controlled, double-blind clinical trial to evaluate the efficacy of polyhexanide for topical decolonization of methicillin-resistant Staphylococcus aureus (MRSA) carriers

**DOI:** 10.1186/2047-2994-4-S1-O6

**Published:** 2015-06-16

**Authors:** C Landelle, E Von Dach, T Haustein, A Agostinho, G Renzi, A Renzoni, D Pittet, J Schrenzel, P François, S Harbarth

**Affiliations:** 1 Infection Control Program, University Hospitals of Geneva and Faculty of Medicine, Geneva, Switzerland; 2 Clinical Microbiology Laboratory, University Hospitals of Geneva and Faculty of Medicine, Geneva, Switzerland; 3 Service of Infectious Diseases, University Hospitals of Geneva and Faculty of Medicine, Geneva, Switzerland; 4 Genomic Research Laboratory, University Hospitals of Geneva and Faculty of Medicine, Geneva, Switzerland

## Introduction

Due to increasing resistance, alternatives to mupirocin and chlorhexidine for decolonization of MRSA carriage need to be evaluated.

## Objectives

To evaluate the efficacy of polyhexanide (Prontoderm^®^) *vs* placebo in eliminating MRSA carriage at day 28 (D28) after the end of treatment.

## Methods

In a 1,900-bed teaching hospital, MRSA-colonized patients were randomized into a double-blind, placebo-controlled superiority trial. Patients were treated with either polyhexanide (antiseptic and surface-active substances; group I) or placebo (only surface-active substances; group P) applied to the anterior nares and skin for 10 days. The primary outcome was MRSA decolonization at D28 assessed by both intention-to-treat ([ITT] responder analysis) and per-protocol (PP) analysis (microbiological follow-up ± 7 days and topical treatment ≥ 5 days). Secondary outcomes included MRSA decolonization according to nasal MRSA carriage, safety and emergence of resistance.

## Results

Of 2590 patients screened, 146 patients (group I, 71; group P, 75) were randomized between January 2011 and July 2014. Primary outcome was missing for 11 (7.5%) patients. ITT analysis showed that 24/71 (33.8%) patients in group I *vs* 22/75 (29.3%) in group P were MRSA-free at D28 (risk difference, 4.5%; 95% CI, -10.6% to 19.5%; *P*=0.56). PP analysis confirmed the results with 19/53 (35.8%) decolonized polyhexanide-treated patients *vs* 17/56 (30.4%) in the placebo arm (risk difference, 5.5%; 95% CI, -12.2% to 23.0%; *P*=0.54). In the subgroup of MRSA nasal carriers, PP analysis showed that 6/15 (40.0%) patients in group I *vs* 2/11 (18.2%) in group P were decolonized (*P*=0.40). Nine serious adverse events occurred in group I *vs* 12 in group P; none was attributable to study medication. Emergence of polyhexanide resistance was not observed.

## Conclusion

This study suggests that under real-life conditions a single polyhexanide decolonization course is marginally effective in eradicating MRSA carriage.

## Disclosure of interest

C. Landelle: None declared, E. Von Dach: None declared, T. Haustein: None declared, A. Agostinho: None declared, G. Renzi: None declared, A. Renzoni: None declared, D. Pittet: None declared, J. Schrenzel: None declared, P. François: None declared, S. Harbarth Grant/Research support from: a peer-reviewed research grant funded by Pfizer, Consultant for: the advisory boards of Destiny Pharma, bioMerieux, Novartis, and DaVolterra

